# Evolution of high-temperature molecular relaxations in poly(2-(2-methoxyethoxy)ethyl methacrylate) upon network formation

**DOI:** 10.1007/s00396-015-3517-8

**Published:** 2015-01-30

**Authors:** Marcin Kozanecki, Marcin Pastorczak, Lidia Okrasa, Jacek Ulanski, Jeong Ae Yoon, Tomasz Kowalewski, Krzysztof Matyjaszewski, Kaloian Koynov

**Affiliations:** 1 Department of Molecular Physics, Lodz University of Technology, Zeromskiego 116, 90-924 Lodz, Poland; 2 Department of Chemistry, Carnegie Mellon University, 4400 Fifth Avenue, Pittsburgh, PA 15213 USA; 3 Max Planck Institute for Polymer Research, Ackermannweg 10, 55021 Mainz, Germany

**Keywords:** Poly(2-(2-methoxyethoxy)ethyl methacrylate), Molecular relaxations, Polymer network, ATRP

## Abstract

Copolymers of 2-(2-methoxyethoxy)ethyl methacrylate (poly(MEO_2_MA)) are regarded as bioinert replacements of poly(*N*-isopropylacrylamide) in some biomedical applications. Networks of poly(MEO_2_MA) of various architecture form thermo-responsive hydrogels. Here, we present dielectric and mechanical spectroscopy studies on segmental motions and network relaxation processes in linear poly(MEO_2_MA) and its networks — bare network and the network grafted with short poly(MEO_2_MA) chains. We show that the α process assigned to the segmental motions of poly(MEO_2_MA) is independent on the polymer topology and the glass transition temperature, *T*_g_, associated with this process equals 235–236 K for all investigated systems. The α′ relaxation observed above *T*_g_ by dynamical mechanical analysis is assigned to the sub-Rouse process. It strongly depends on the polymer network architecture and slows down by four orders of magnitude upon network formation.

**Electronic supplementary material:**

The online version of this article (doi:10.1007/s00396-015-3517-8) contains supplementary material, which is available to authorized users.

## Introduction

Copolymers of 2-(2-methoxyethoxy)ethyl methacrylate (MEO_2_MA) and oligo(ethylene glycol) methacrylate (OEGMA) [1–9] constitute an interesting alternative to poly(*N*-isopropylacrylamide) (PNIPAAM), the most often studied temperature-responsive polymer for biomedical applications [10, 11]. Importantly, in contrast with the relatively cytotoxic PNIPAAM [12], poly(MEO_2_MA-*co*-OEGMA) copolymers appear to be non-toxic and non-immunogenic [13], as both monomers are analogues of bioinert poly(ethylene glycol) (PEG). Recently, MEO_2_MA was used in the synthesis of stimuli-responsive polymer brushes [14], chemically cross-linked hydrogels with various network architecture for drug delivery [4, 15] and magnetic microgels loaded with Fe_3_O_4_ for targeted drug delivery [5]. Furthermore, a copolymer hydrogel designed for harvesting of gene modified cells or tissue engineering was synthesized from MEO_2_MA, which was additionally mechanically strengthened by copolymerization with 2-vinyl-4,6-diamino-1,3,5-triazine [13]. These examples of application of MEO_2_MA-based materials point out to their considerable promise as thermo-responsive systems for pharmaceutical and biomedical industries.

**Fig. 1:**
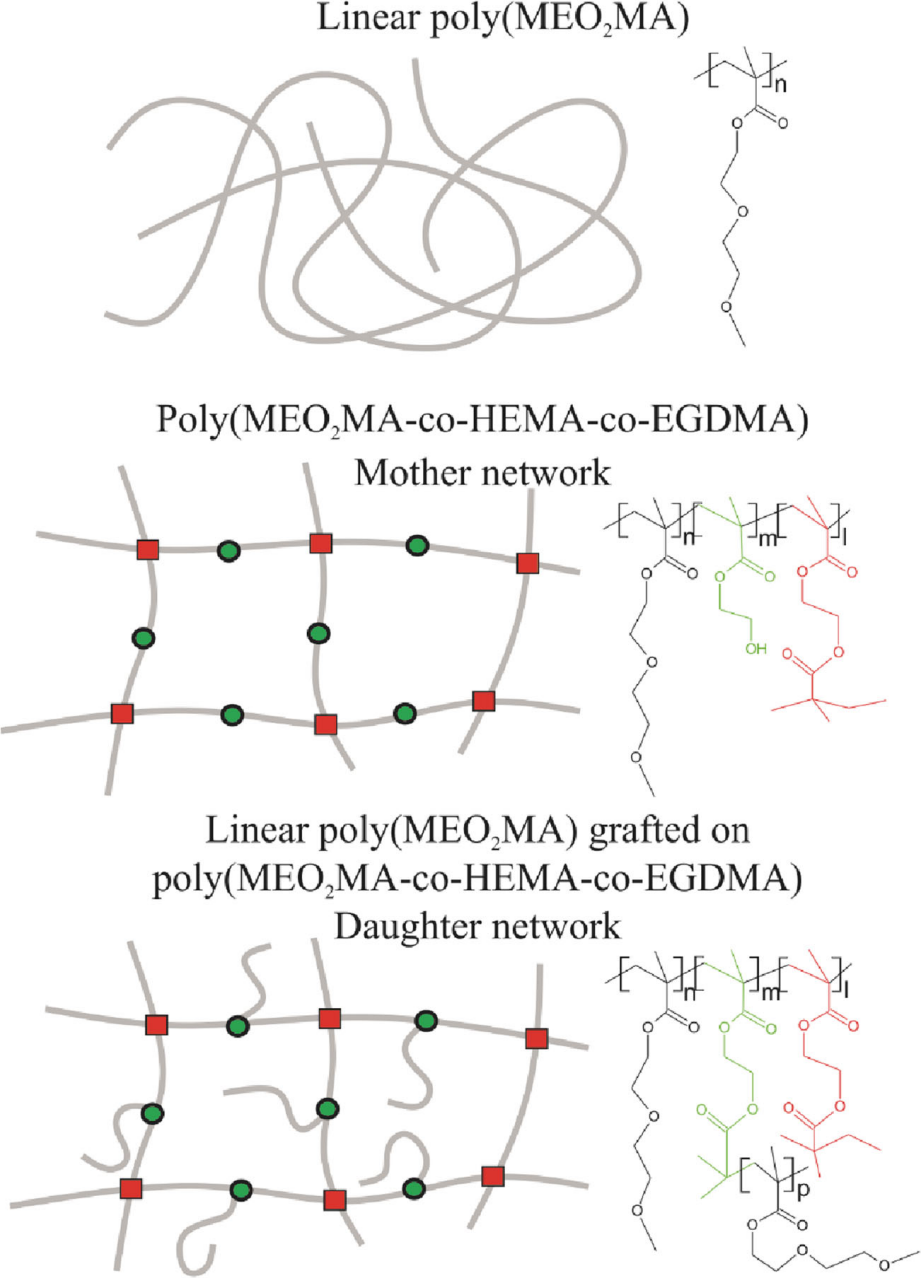
Structure of the investigated materials; *black* elements represents linear poly(MEO_2_MA), *green* — grafting centres (HEMA) and *red* — cross-links (EDGMA). In a case of networks represents the relative ratio of particular components, respectively, *n* = 500, *m* = 5, *l* = 5

In this paper, we focus on the relaxation processes observed above glass transition temperature (*T*_g_) in poly(MEO_2_MA)-based networks of various architecture. Precisely, the objects of our studies were dry-state poly(MEO_2_MA)-based systems of the following topologies: (i) pure linear poly(MEO_2_MA); (ii) cross-linked network (referred to as “mother network”) of copolymer poly(MEO_2_MA-*co*-HEMA-*co*-EGDMA), where HEMA is 2-hydroxyethyl methacrylate and EGDMA is ethylene glycol dimethacrylate used as a cross-linking agent and (iii) “daughter network” with dangling chains formed by grafting linear poly(MEO_2_MA) from the “mother network”. All materials were synthesized using atom transfer radical polymerization (ATRP) [16, 17], which assured their well-defined nature, with relatively narrow distributions of chain lengths between cross-links and of lengths of grafted dangling chains. Chemical structures, as well as topologies of investigated materials and their labelling, are schematically presented in Fig. 1. Investigations on dried networks based on poly(MEO_2_MA) is a starting point for further studies on hydrogels based on these systems.

Relaxation processes in poly(MEO_2_MA) materials were investigated by two complementary techniques: dielectric relaxation spectroscopy (DRS) and dynamic mechanical analysis (DMA). Interpretation of the obtained results is supported by differential scanning calorimetry (DSC).

## Experimental

### Samples

#### Preparation of linear poly(MEO_2_MA)

2-(2-Methoxyethoxy)ethyl methacrylate (MEO_2_MA, 3.0 mL, 16 mmol), *N*,*N*,*N*′,*N*″,*N*″-pentamethyldiethylene triamine (34 μL, 0.16 mmol) and 3 mL of anisole were added to a 10-mL Schlenk flask, and the reaction mixture was degassed by three freeze–pump–thaw cycles. Then, CuBr (22 mg, 0.16 mmol) was added to the frozen reaction mixture under nitrogen flow. One more freeze–pump–thaw cycle was repeated, and the flask was back filled with nitrogen. N_2_-bubbled ethyl 2-bromoisobutyrate (EBiB, 24 μL, 0.16 mmol) was injected to the flask under N_2_ flow, and the flask was placed in an oil bath preheated to 333 K. The progress of the reaction was followed by gas chromatography (GC) and gel permeation chromatography (GPC) analyses. When a targeted molecular weight was obtained, the reaction was stopped by opening the flask and exposing the contents to air. The polymer with *M*_n_ = 9700, *M*_w_/*M*_n_ = 1.26 was prepared (GPC in tetrahydrofuran (THF) eluent, polymethylmethacrylate (PMMA) standard).

#### Preparation of mother network

MEO_2_MA (20.0 mL, 108 mmol), 2-hydroxyethyl methacrylate (HEMA, 132 μL, 1.08 mmol), ethylene glycol dimethacrylate (EGDMA, 205 μL, 1.08 mmol), tris(2-pyridylmethyl)amine (TPMA, 4.7 mg, 0.016 mmol), CuBr_2_ (1.2 mg, 0.0054 mmol) and anisole (2 mL) were added to a 50-mL cylindrical jar with the diameter of 5 cm. The pre-gel reaction mixture was degassed by bubbling for more than 30 min. Then, tin(II) 2-ethylhexanoate (Sn(EH)_2_, 13.2 mg, 0.0326 mmol) and N_2_-bubbled EBiB (32 μL, 0.218 mmol) were injected. The molar ratio of MEO_2_MA:HEMA:EGDMA:EBiB:TPMA:CuBr_2_:Sn(EH)_2_ was 500:5:5:1:0.075:0.025:0.15. The jar was placed in an oil bath preheated to 333 K, and the nitrogen stream was maintained for 3 h. After 3 h, additional 10 mg (0.025 mmol) of Sn(EH)_2_ was added. The stirring and the nitrogen flow were stopped for a bubble-free gel formation. After 2 h, gel was formed and the reaction was maintained for additional 12 h (conversion: 90 % by GC). The prepared disc-shaped gel was purified and sliced into rectangular pieces (*ca*. 2 cm × 2 cm × 0.3 cm). The purification was performed by extraction with a plenty amount of acetone and replacing the acetone for more than five times.

#### Introducing ATRP-initiating sites into the mother network

ATRP-initiating sites were introduced to gels by the condensation reaction between hydroxyl groups on the gels with α-bromoisobutyryl bromide (BIBB). To pieces of gel swollen in dry THF, was added triethylamine (TEA, 3.5 mL, *ca*. 60-fold excess compared to the amount of HEMA (*ca*. 0.06 mL, 0.45 mmol) in the used gel). After the permeation of TEA throughout the gel, BIBB (3.0 mL, *ca*. 50-fold excess compared to the amount of HEMA) was injected into the flask. The reaction mixture was stirred for 1 day. The resulting gels were purified by sequential extractions with THF, THF/water mixture, water, water/acetone mixture, and finally acetone for 1 day. The gels modified with ATRP-initiating sites were used as macroinitiators for the subsequent grafting-from reactions.

#### Preparation of daughter network

Gels containing dangling grafted chains were prepared by conducting a “graft-from” polymerization using the gels containing ATRP-initiating sites. MEO_2_MA (10.0 mL, 54 mmol) was injected into acetone-swollen gels and aged for 2 h. After the diffusion of the monomer throughout the gel, acetone and air were removed under vacuum at room temperature. Since the boiling point of MEO_2_MA under vacuum (2 mmHg) is above 333 K, the amount of MEO_2_MA lost from the flask during this step was negligible. In a separate Schlenk flask, a catalyst stock solution containing CuBr (39 mg, 0.27 mmol), and 4,4′-dinonyl-2,2′-bipyridine (264 mg, 0.54 mmol) in 10 mL of anisole was prepared. The catalyst stock solution was transferred under nitrogen flow to the Schlenk flask, containing MEO_2_MA diffused gels, and the flask was kept in a refrigerator (275 K) for ∼3 h. After the reactants penetrated to the centre of the gels, EBiB (80 μL, 0.54 mmol) was injected in order to independently follow the molecular weight of grafted chains. The reaction mixture was heated to 323 K. Samples of the reaction mixture were taken periodically by syringe to measure the molecular weight of unattached polymer chains grown from the “free” initiators by GPC, using linear PMMA standards. The molecular weight of the free polymers was assumed to be identical to that of the grafted dangling chains: Mn = 1.21 × 10^4^ (DP 64), Mw/Mn = 1.26. When a target molecular weight was reached, the reaction was stopped by opening the reaction mixture to air and dilution with acetone. The grafted gel was purified by several extractions with acetone. Hydrogel samples in equilibrium swollen state were dried, initially under ambient conditions and finally at least 24 h under vacuum.

### Measurements

Differential scanning calorimetry (DSC) measurements were performed using a Mettler DSC-30 calorimeter. The cooling/heating rate was 10°/min. The glass transition temperature, *T*_g,_ was determined from the second heating run as a middle point of the d*H*/d*t* step in the DSC trace.

The dielectric relaxation spectroscopy (DRS) was carried out using the CONCEPT 80 dielectric spectrometer (Novocontrol® GmbH, Germany). All samples were prepared in a form of films with thickness about 100 μm. The samples were sandwiched between two polished brass electrodes covered with a thin layer of gold (20 mm in diameter) and placed inside the temperature-controlled sample cell. The complex permittivity ε*(*f*) = *ε*′(*f*) − *iε*″(*f*) was determined in the frequency *f* range from 0.03 to 10^6^ Hz and in the temperature range from 173 to 348 K. Temperature was controlled using a nitrogen gas cryostat with the stability better than 0.1°. Dielectric isothermal spectra were analysed using the WinFIT software (Novocontrol® GmbH, Germany), and the relaxation times (*τ*) of particular processes at various temperatures were determined using Havriliak–Negami type function:

1 $$ {\varepsilon}^{*}\left(\omega \right)={\displaystyle \sum_k}{\varepsilon}_{rk}^{*}+{\varepsilon}_{\sigma}^{*}={\varepsilon}_{\infty k}+{\displaystyle \sum_k}\frac{\varepsilon_{0k}-{\varepsilon}_{\infty k}}{{\left(1+{\left(i\omega {\tau}_k\right)}^{\alpha_k}\right)}^{\beta_k}}-i{\left(\frac{\sigma_0}{\varepsilon_0\omega}\right)}^s $$

where *ε*_*σ*_ and *ε*_*rk*_ correspond to the contributions related to ionic conductivity (*σ*_0_) and *k* relaxation process. Symbols *ε*_0_, *ε*_0*k*_, *ε*_∞*k*_ denote, respectively, vacuum permittivity, static (low frequency) permittivity and permittivity at the infinite frequency. The exponents *α*_*k*_ and *β*_*k*_ describe the asymmetry and broadness of a band corresponding to *k* process. Parameter *s* generally equals 1. Relaxation times of particular processes at various temperatures were determined from the frequency domain plots of dielectric permittivity, using both the positions of maxima in *ε*″ curves and the inflexion points of *ε*′ curves.

The dynamic mechanical analysis (DMA) was performed using a RMS 800 rheometer (Rheometrics). Oscillatory shear deformation was applied with controlled deformation amplitude, which was kept in the range of the linear viscoelastic response. Plate–plate geometry was used with plate diameter of 6 mm and sample thickness of *ca.* 1 mm. Experiments were performed in a dry nitrogen atmosphere in the temperature range between 243 and 303 K or in an air atmosphere at temperatures above 303 K. The DMA spectra (values of storage (*G*′) and loss (*G*″) shear modulus, as well as loss tangent (tan *δ* = *G*″/*G*′) as a function of radial frequency *ω* in the range from 0.1 to 100 rad/s) were obtained at various constant temperatures maintained to within ±0.1°. Samples were kept for 2 min at each temperature before the measurement.

Because the relaxation times *τ*_e_ estimated from the maxima of dielectric loss (*ε*″) or inflexions of permittivity (*ε*′) correspond directly to the retardation times *τ*_*j*_ estimated from the maxima of mechanical compliance *j*″(*ω*), the storage (*G*′) and loss (*G*″) moduli were converted into the compliances *j*′ and *j*″ according to the following equations [18]:

2a $$ j^{\prime }=\frac{G^{\prime }}{G{\prime}^2+G{^{{\prime\prime}}}^2}, $$

2b $$ j^{{\prime\prime}}=\frac{G^{{\prime\prime}}}{G{\prime}^2+G{^{{\prime\prime}}}^2}. $$

The mechanical isothermal spectra were analysed with the aid of the WinFIT software (Novocontrol® GmbH, Germany) analogously to dielectric ones using the Havriliak–Negami formalism. If the *j*″(*ω*) signal was too weak to determine *τ*_*j*_ (it was the case only for linear polymer), the relaxation times *τ*_*G*_ obtained from loss modulus as well as times *τ*_tan_ corresponding to maxima of mechanical loss tangent tan *δ*(*ω*) were taken into account and the retardation times were estimated according to the formula commonly used for the single Debye process [18]:

3a $$ {\tau}_j=\frac{G_U}{G_R}{\tau}_G, $$

3b $$ {\tau}_j={\left(\frac{G_U}{G_R}\right)}^{1/2}{\tau}_{\tan }, $$

where *G*_*U*_ and *G*_*R*_ denote mechanical modulus at time 0 and ∞, respectively.

## Results and discussion

### Calorimetric measurements

**Fig. 2:**
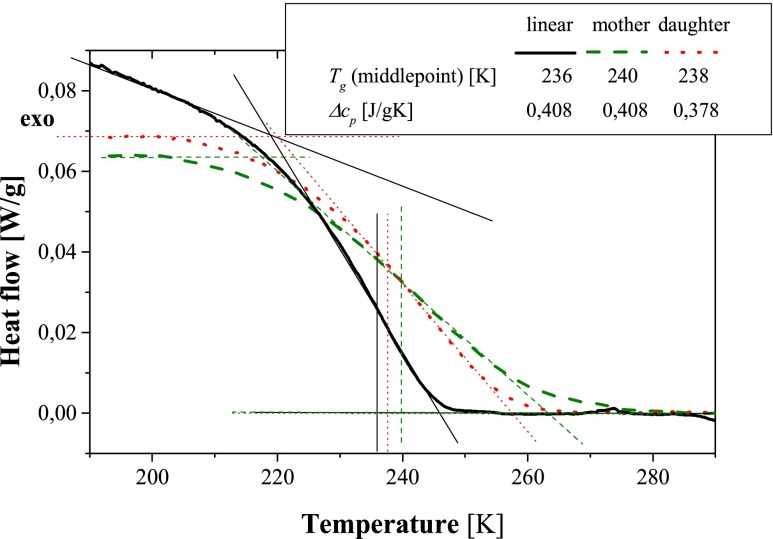
DSC thermograms for linear and cross-linked poly(MEO_2_MA)-based materials

The DSC thermograms of linear poly(MEO_2_MA) and of the mother and daughter networks are presented in Fig. 2. The glass transition temperature of the linear polymer, determined as a transition midpoint, was equal to 236 K. The glass transition temperatures determined in a similar manner for the mother and daughter networks were only slightly higher (respectively, 240 and 238 K). Such only modest increase of *T*_g_ in cross-linked networks in comparison with the linear polymer can be explained by the limited impact of low cross-linking densities on the onset of segmental mobility of polymer chains. Given their minuscule content (*ca.* 1 %), the presence of HEMA and EGDMA comonomers in the networks is not expected to have a significant impact on the observed glass transition temperatures. It is important to point here that studied samples were stored at room temperature, which is well above their *T*_g_ determined by DSC. Henceforth, the samples used for mechanical analysis and dielectric studies were initially in a stress-relaxed state.

The Δ*c*_*p*_ value for linear poly(MEO_2_MA) was estimated to be equal to 0.408 J/g · K. Comparison of DSC thermograms acquired for mother and daughter networks shows that the Δ*c*_*p*_ is practically independent on network structure: 0.408 and 0.378 J/g · K for mother and daughter network, respectively. These results are not surprising given low cross-linking density of investigated networks (below 1 %).

### Dielectric measurements

**Fig. 3:**
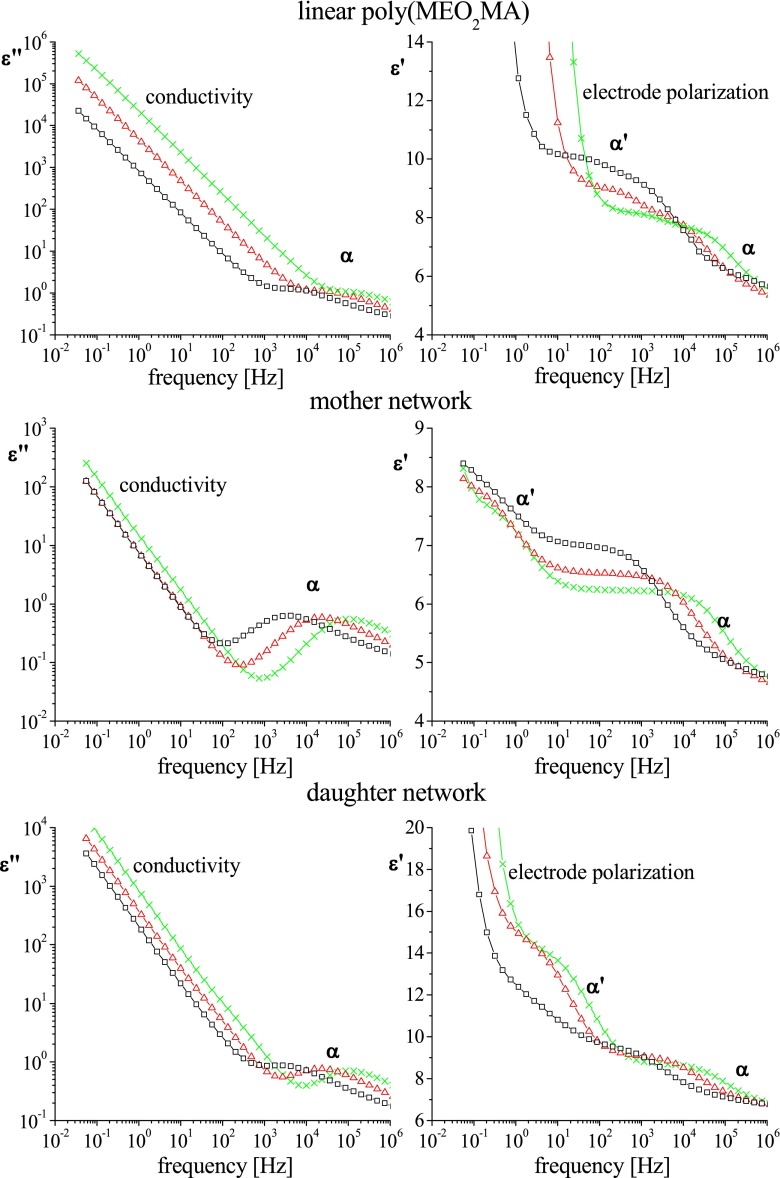
Dependences of dielectric loss (*ε*″) and dielectric constant (*ε*′) versus frequency for poly(MEO_2_MA)-based samples of different architecture at various temperatures: 305 K (*squares*), 323 K (*triangles*) and 341 K (*crosses*)

For all studied materials, the DRS spectra showed above the *T*_g_ the presence of two relaxation processes marked as α and α′. Additionally, as one can see in the Figure A1 in Online Resource 1, in the glassy state (below *T*_g_), three secondary processes were observed, which are not a subject of this paper. Temperature dependences of particular secondary processes are very similar in all investigated samples. Comparison of frequency dependences of dielectric loss (*ε*″) and dielectric constant (*ε*′) for various samples at some arbitrarily chosen temperatures higher than calorimetrically determined *T*_g_ is presented in Fig. 3.

The temperature dependences of all observed dielectric processes are shown together with DMA results in the Arrhenius plot in Fig. 7. The α process is assigned to the segmental motions and, in literature, is sometimes referred to as the dynamic glass transition. This type of process can be described by the Vogel–Fulcher–Tammann (VFT) equation [19–21]:

4 $$ \tau (T)={\tau}_0 \exp \left(\frac{D_0{T}_v}{T-{T}_v}\right) $$

where *τ*_0_ is the relaxation time in the limit of high temperatures, *D*_0_ is the fragility index describing a deviation of temperature dependence of *τ* from the Arrhenius law and *T*_*v*_ is the so-called Vogel temperature, which for polymers is typically 30–70 K lower than *T*_g_ and sometimes is identified as “ideal” glass transition temperature [22]. The values of these parameters obtained by fitting DRS results to Eq. (4) are presented in Table 1. The free volume (*φ*) in *T*_100_ (the temperature at which the relaxation time of the α relaxation process reached 100 s is usually very close to *T*_g_ determined calorimetrically) was estimated using the relationship proposed by Doolittle [23, 24]:

**Table 1:** Comparison of basic parameters of VFT equation determined for α processes for poly(MEO_2_MA)-based materials with various architecture

	Linear poly(MEO_2_MA)	Mother network	Daughter network
*T*_*v*_ [K]	160 ± 1	154 ± 3	161 ± 3
*τ*_0_ [s]	(1.70 ± 0.01)*10^−12^	(1.70 ± 0.03)*10^−12^	(3.42 ± 0.04)*10^−12^
*D*_0_	15.0 ± 0.4	17 ± 1	14.7 ± 0.8
*T*_100_[K]^a^	235 ± 1	235 ± 1	236 ± 1
*φ*/*B* [%]	3.2 ± 0.3	3.2 ± 0.5	3.2 ± 0.5
*α*_*f*_ × 10^−4^ [K^−1^]	4.18 ± 0.09	3.9 ± 0.1	4.2 ± 0.1
*m*	43 ± 3	40 ± 6	42 ± 5
*E*_a_′(*T*_100_) [kJ mol^−1^]	196 ± 16	183 ± 31	195 ± 33

^a^ *T*_100_ stands for the temperature at which relaxation time of α process is equal to 100 s. For α relaxation, this corresponds to the glass transition temperature determined calorimetrically

5 $$ \frac{\phi }{B}=\frac{\left({T}_{100}-{T}_v\right)}{D_0{T}_v} $$

where *B* is a parameter close to unity. A thermal expansion coefficient (*α*_*f*_) at *T*_100_ was determined according to the following formula:

6 $$ \frac{\alpha_f}{B}=\frac{\left(\frac{1}{v}\right){\left(\frac{\partial v}{\partial T}\right)}_p}{B}=\frac{1}{D_0{T}_v} $$

In addition, the dynamic fragility factor *m* was calculated, using the following equation [25]:

7 $$ m=\frac{D_0{T}_v{T}_{100}}{ln10\cdot {\left({T}_{100}-{T}_v\right)}^2} $$

According to the Angell’s concept [26], the “fragile” liquids (low *m* values) exhibit more rapid changes of relaxation times than the “strong” liquids (high *m* values) in the temperature range rising through the glass transition region.

We have calculated also the apparent activation energy *E*_a_′ at *T*_100_ using the following formula [27]:

8 $$ {E}_{\mathrm{a}}^{\prime}\left({T}_{100}\right)=\frac{R{D}_0{T}_v}{{\left(1-\frac{T_v}{T_{100}}\right)}^2} $$

where *R* is the universal gas constant and is equal 8.314 J mol^−1^ K^−1^.

Comparison of values listed in Table 1 points to the close similarity of the parameters of the α relaxation process between all investigated samples. In particular, the values of *T*_100_ temperature, which is commonly associated with the glass transition temperature, were all within 235–236 K. These values, in turn, are close to the calorimetrically determined glass transition temperatures (cf. Fig. 2), pointing to the good consistency between calorimetric and DRS measurements.

Lack of dependence of the parameters of the α process on polymer topology evident from the results shown in Table 1 can be explained by low cross-linking density of the samples. It is also consistent with past reports of insensitivity of the α process of other hydrophilic vinyl polymers to chemical cross-linking [28].

While the α process could be unambiguously associated with the segmental motion corresponding to the glass transition temperature, the origin of the α′ process occurring within the similar temperature range but at much lower frequencies is not clear. In contrast with the α process, which was insensitive to the polymer topology, the α′ process was faster in the linear polymer. Given the absence of permanent dipole moment along the main chain, the α′ cannot be related to the so-called normal mode. For this reason the similar relaxation above *T*_g_ found by Carsi et al. [25] in dielectric spectra for cross-linked CEOEMA was attributed by the authors to Maxwell–Wagner–Sillars (MWS) relaxation arising from a long-distance charge transport and polarization at the interfaces taking place in the bulk in heterogeneous systems.

Since MWS is an effect strictly related to polarization, a process corresponding to it should not have any contribution to a mechanical relaxation spectrum. Thus, in order to elucidate the origin of the α′ process, the poly(MEO_2_MA) systems were further characterized by DMA.

### Dynamic mechanical analysis

**Fig. 4:**
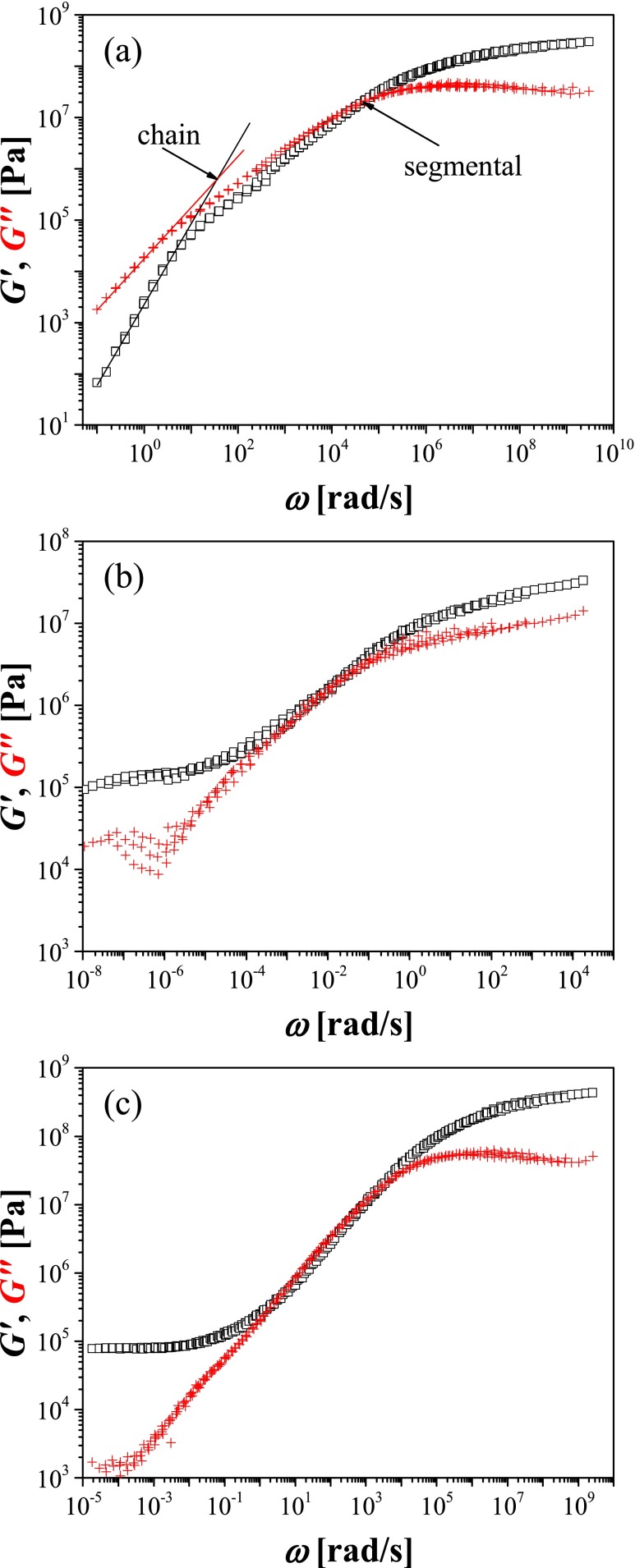
Master curves created based on DMA spectra for linear poly(MEO_2_MA) (**a**), mother (**b**) and daughter (**c**) networks. *Squares* correspond to *G*′, while *crosses* to *G*″. The curves are constructed at reference temperature 293 K. *Red line* in panel **a** corresponds to the slope ∼*ω* ^0.5^ which is a characteristic for the chain relaxation

The experimentally accessible frequency range covered by the classical DMA technique used in this work is rather limited, extending from 0.1 to 100 rad/s. The common way of expanding the dynamical range of time-dependent mechanical analysis is based on the time–temperature superposition and involves construction of master curves for the real *G*′ and imaginary *G*″ part of the complex shear modulus at a given reference temperature. The master curves are created by shifting the data recorded at various temperatures along the frequency coordinate. Next, relaxation processes are identified by the crossover of *G*′(*ω*) and *G*″(*ω*) curves, and the temperature dependence of the corresponding relaxation times is evaluated using the shift factors. Such master curve constructed for the linear poly(MEO_2_MA) is shown in Fig. 4a. The crossover of *G*′(*ω*) and *G*″(*ω*) in the high-frequency range corresponds to the segmental relaxation and, as discussed later, the temperature dependence of the respective relaxation time closely follows that obtained by DRS (cf. Fig. 7).

The crossover at low frequencies (Fig. 4a) corresponds to the chain relaxation. Interestingly, the temperature dependence of the chain relaxation time follows closely that of the α′ processes observed with DRS. This issue will be discussed in more details below. In contrast to the neat polymer, the master curves constructed based on DMA spectra collected for poly(MEO_2_MA)-based networks are not so unambiguous for two main reasons. First of all, the mechanical measurements at temperatures below 250 K were not possible in the case of cross-linked materials, because of loss of contact between the sample and the rheometer plates (the sample became too stiff). Secondly, the time–temperature superposition cannot be always applied to cross-linked networks. We found that the temperature dependences of the shift factors obtained for both networks did not conform well to the Williams–Landell–Ferry (WLF) equation. Furthermore, in all cases, small vertical shifting was required to construct smooth “master curves”. For these reasons in what follows, we used directly the experimentally measured spectra *G*′(*ω*) and *G*″(*ω*), in the frequency range 0.1 to 100 rad/s at various temperatures, to evaluate the temperature dependence of the relaxation times in all studied systems. As the DMA experiments with the cross-linked networks could not be performed below 250 K, the α relaxation process was analysed only for the linear polymer.

**Fig. 5:**
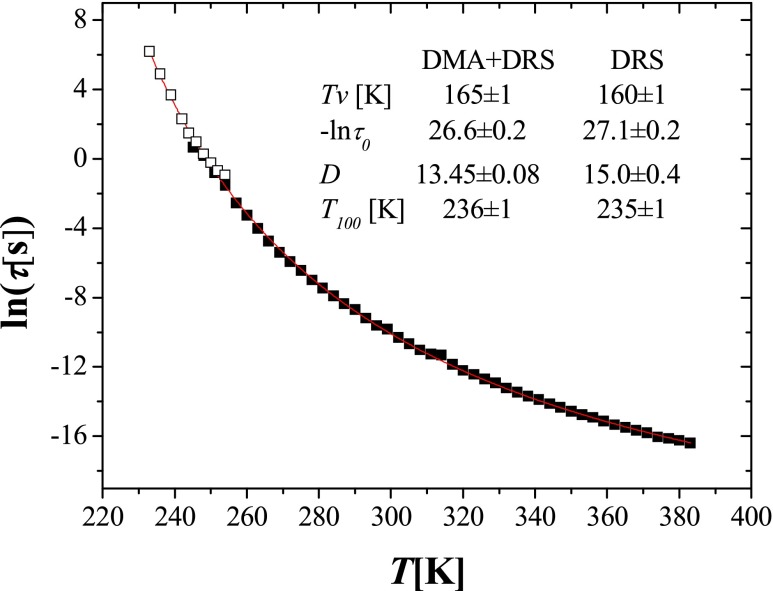
Comparison of relaxation times determined for the α process in linear poly(MEO_2_MA) by DRS (*full symbols*) and DMA (*open symbols*) at various temperature. Additionally, the parameters of VFT equation fitted based on DMA and DRS results are compared with the values obtained based only on DRS data analysis

As it was mentioned in the “Experimental” part, the dielectric relaxation time *τ*_*e*_ corresponds directly to the mechanical *τ*_*j*_. Due to very weak amplitude of the band related to the α process in the *j*″(*ω*) representation, the retardation time *τ*_*G*_ from *G*″(*ω*) was determined and then recalculated into *τ*_*j*_ according to the Eq. (3a). Values of *G*_*U*_ and *G*_*R*_ assumed for particular samples and processes are listed in Table 2. Although Eq. (3a) is commonly used for the single Debye process, we decided to use it for estimation of the retardation times of the α process, since it was not possible to determine the retardation times directly from the analysis of the mechanical compliance spectra. Figure 5 shows a good agreement of the experimental points for α process in linear poly(MEO_2_MA) obtained by DRS and DMA.

**Table 2:** Values of *G*_*U*_ and *G*_*R*_ of mechanical modulus at time 0 and ∞, respectively, used for calculation of relaxation times from DMA results

	Linear poly(MEO_2_MA)		Mother network	Daughter network
	α	α′		
*G*_*R*_ [Pa]	4 · 10^6^	2.5 · 10^4^	5 · 10^5^	4 · 10^4^
*G*_*U*_ [Pa]	2 · 10^8^	4 · 10^6^	3 · 10^7^	4 · 10^6^

**Fig. 6:**
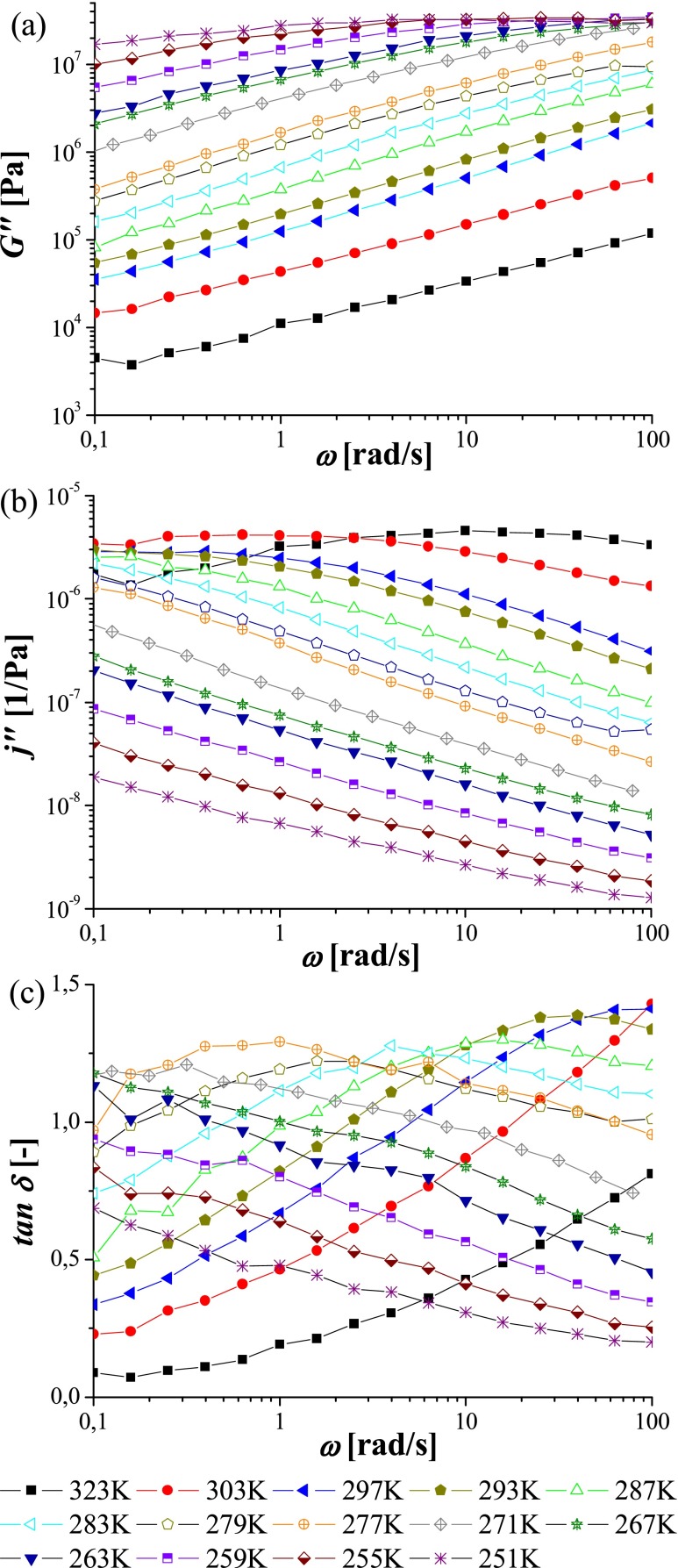
DMA spectra acquired for daughter network: **a** *G*″(*ω*), **b** *j*″(*ω*) and **c** tan *δ*(*ω*)

Fortunately, the range of frequencies and temperatures accessible for the DMA experiments covers well the range of the α′ process which is of particular interest here. Moreover, the amplitude of the band in the *j*″(*ω*) dependences, corresponding to the α′ process is sufficient to directly determine the retardation time *τ*_*j*_ by means of the Havriliak–Negami function. Additionally, to widen the analytical range, the *τ*_tan_ was also determined from the maximum of tan *δ* and convert to *τ*_*j*_ according to Eq. (3b). Figure 6 shows the frequency dependences *G*″(*ω*), *j*″(*ω*) and tan *δ(ω*) determined for the daughter network.

**Fig. 7:**
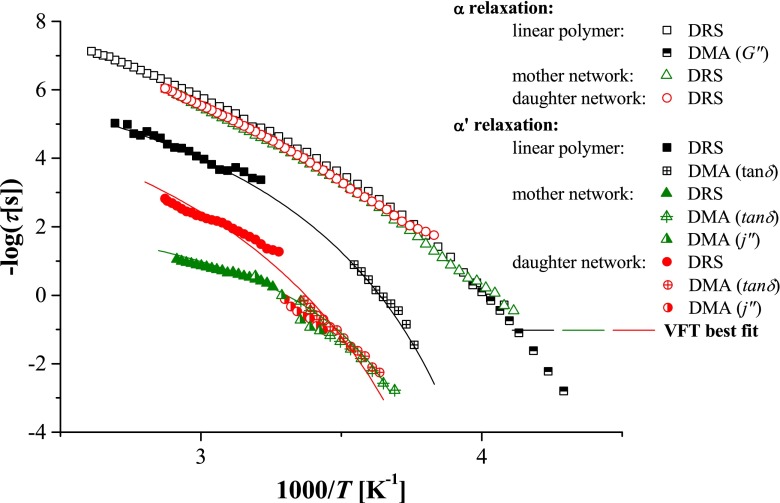
Activation map for poly(MEO_2_MA)-based materials differing on polymer architecture, constructed basing on DRS and DMA results

In order to compare the DRS and DMA results, an activation map for α′ relaxation was constructed as shown in Fig. 7. The α′ process was also fitted by the VFT equation similarly to the α relaxation, be justified by the fact that the material is in a viscoelastic state. The parameters of VFT equation obtained by fitting the α′ relaxation data for all samples are listed in Table 3.

**Table 3:** Comparison of basic parameters of VFT equation determined for α′ processes for poly(MEO_2_MA)-based materials with various architecture

	Linear poly-(MEO_2_MA)	Mother network	Daughter network
*T*_*v*_ [K]	219 ± 4	240 ± 2	218 ± 2
*τ*′_0_ [s]	(15.2 ± 0.7)*10^−9^	(1.01 ± 0.4)*10^−3^	(25.1 ± 0.7)*10^−9^
*D*′_0_	4.6 ± 0.9	1.8 ± 0.2	6.3 ± 0.3
*T*_100_ [K]^a^	263 ± 4	278 ± 3	281 ± 2
*E*_a_′ (*T*_100_) [kJ/mol]	297 ± 98	197 ± 68	228 ± 49

^a^ *T*_100_ stands for the temperature at which relaxation time of α′ process is equal 100 s

Good agreement between DMA and DRS results for α′ relaxation, shown in Fig. 7, and in particular the fact that the temperature dependence of DMA and DRS relaxation times could be fitted to VFT equation using the same parameters are strong indications that the α′ relaxation observed by both techniques is related to the same process. Therefore, the mechanical manifestation of this process is a strong argument against the possibility that it is directly related to the MWS effect; however, the response seen in the DRS spectra can be due to an increase of ionic mobility triggered by molecular relaxation.

The presented above evidence that the α′ process is related to molecular relaxations leads to the question about its mechanism. According to Plazek [29, 30], for linear polymers, the α′ process may be assigned to the sub-Rouse and the Rouse modes. On a time scale longer than the characteristic for segmental relaxation (α process) the diffusion properties of the whole chains appear. They are usually described as purely entropic Rouse modes observed by mechanical relaxation spectroscopy [31, 32]. For the polymers with a constant dipole moment along the main chain, discussed processes are observable also by dielectric spectroscopy and called normal mode.

In some polymers, an additional so-called sub-Rouse mode located between the normal mode and segmental relaxation was observed. For a long time, it had been believed that the sub-Rouse mode in B- and C-type polymers may be measured only by mechanical methods. Recently, Paluch et al. [33] showed that activity of the sub-Rouse mode depends on the ordering of dipole moment in polymer chain and on the degree of tacticity. The authors demonstrated that the sub-Rouse mode may be observable by DRS method in A-type polymer like polyisoprene as well as in B-type polymer – polybutylene. Additionally, they demonstrated that the sub-Rouse mode may be observed by DRS in head-to-head polypropylene as a result of a relatively high correlation between the normal dipole moments in this polymer, while in atactic polypropylene with random orientation of dipole moments, the sub-Rouse mode is undetectable by dielectric measurements. Wang et al. [34] used 2D correlation DRS to study glass–rubber transition region in polybutylene. They showed that this technique may be a powerful tool to study Rouse and sub-Rouse modes in B-type polymers. Our calculations (see also Figure A2 in Online Resource 1) indicate that the projection of the dipole moment vector on the main chain is nonzero in the case of helical topology of poly(MEO_2_MA) macromolecule (trans-gauche conformation). Such arrangement of MEO_2_MA units may result in local correlation of elementary dipole moments and finally lead to the activity of sub-Rouse modes in DRS.

Another possible explanation of an origin of the observed α′ processes relates to the relaxation time *τ*_*σ*_ characteristic of ionic conductivity resulting from impurities in the studied samples. We determined the relaxation time *τ*_*σ*_ from the crossing points of the *ε′*(*f*) and *ε″*(*f*) plots. The plots of log(*τ*_*σ*_) versus 1/*T* are shown in Fig A5 in the Online Resource 1 file. The comparison of such obtained dependences with the α′ processes shows that only in the highest temperature range (above ca. 320–325 K) the ionic conductivity is correlated with the α′ process, while in the range of lower temperatures, the discussed dependences bifurcate. Hence, we conclude that the α′ process originates from molecular relaxations which above around 320 K alter the mobility of ionic impurities. Recently, similar correlation between polymer chain dynamics and ions mobility was reported by Zardalidis et al. who studied archetypal polymer electrolyte (PEO)_x_LiCF_3_SO_3_ [35]. Interpretation presented herein is additionally supported by the conformability of the DRS and DMA results as well as by the fact that the α′ process is well discernible in the *ε*′ spectra shown in Fig. 3.

An argument in favour of the possibility that the α′ process is related to the sub-Rouse mode is provided also by the observation of the significant (at least three orders of magnitude) increase of its relaxation time upon cross-linking (Fig. 7, mother network sample). The *T*′_100_ transition temperature, analogous to *T*_*100*_ determined by DRS and corresponding to the temperature at which the relaxation time of α′ process reaches 100 s, is also considerably higher (>12 K). Such slowing down could be explained by the restrictions imposed on the sub-Rouse modes of poly(MEO_2_MA) chains by the crosslink (EGDMA) centres. This effect is also consistent with the observed increase of activation energy (see Table 3). The other argument in support of our interpretation of the α′ process is the fact that for all samples studied herein, storage modulus *G*′(*ω*) scales as *ω* ^0.5^ above the rubbery plateau region. Presence of such power law scaling is an indication of a Rouse chain mode according to the Rouse theory [36, 37].

The low-temperature part of the α′ process curve in the relaxation map of the daughter network (up to ∼300 K) overlaps with the curve for mother network (Fig. 7), suggesting that the presence of dangling chains has little effect on the onset of sub-Rouse modes. Accordingly, the *T*′_100_ temperatures and activation energies for both types of networks are also similar. Interestingly, however, at higher temperatures, the curve for the daughter network deflects upwards, pointing to the acceleration of the α′ process in comparison with the mother network. Such acceleration may be the evidence that dangling flexible chains act as a “self-plasticizer”, facilitating long-range motion of network segments otherwise restricted by the presence of cross-links [38]. Such self-plasticization by dangling chains can be also inferred from comparison of mechanical properties of the mother and daughter networks in the rubbery region (e.g. at 313 K), with the daughter network being considerably softer (∼40 kPa vs. ∼100 kPa).

## Conclusion

Characterization of high-temperature relaxation processes in linear poly(MEO_2_MA) and two poly(MEO_2_MA)-based networks (bare and decorated by poly(MEO_2_MA) dangling chains) was the main goal of the presented studies. The performed investigations have shown that the determination by DSC glass transition temperature as well as the related primary α relaxation process are very similar in all materials and practically not affected by the network formation. These similarities may be explained by rather low density of cross-links in the considered networks.

The α′ process observed in the DMA spectra most probably originates from the sub-Rouse modes. This process exhibits pronounced dependence on the network architecture: cross-linking significantly slows it down (by at least three orders of magnitude). In the similar range of temperatures and frequencies, we observed the α′ process by DRS which can be assigned to the ionic mobility. It was also found that above 320 K the dielectric and mechanical α′ processes are strongly correlated with each other in temperature and frequency. Thus, we postulate that in that temperature range, the ionic mobility is stimulated by the molecular relaxations of the polymer chains that results in coupling of both processes.

## Electronic supplementary material

Below is the link to the electronic supplementary material.

ESM 1 (DOCX 543 kb)
