# Supplementary material for: Evolution of high-temperature molecular relaxations in poly(2-(2-methoxyethoxy)ethyl methacrylate) upon network formation
Source: Colloid Polym Sci. 2015 Jan 30;293(5):1357–67. doi: 10.1007/s00396-015-3517-8 (PMC4544547; doi:10.1007/s00396-015-3517-8)
Supplement: Supplementary file 1 — (DOCX 543 kb) [file 396_2015_3517_MOESM1_ESM.docx]

Supplementary data

**Evolution of high-temperature molecular relaxations in poly(2-(2-methoxyethoxy)ethyl methacrylate) upon network formation**

Colloid and Polymer Science

M. Kozanecki*, M. Pastorczak, L. Okrasa, J. Ulanski, J.-A. Yoon, T. Kowalewski, K. Matyjaszewski, K. Koynov

*Corresponding author: e-mail: marcin.kozanecki@p.lodz.pl; telephone: +4842 6313205; fax +48426313218; Department of Molecular Physics, Lodz University of Technology, Zeromskiego 116, 90-924 Lodz, Poland

**Fig. A1** Examples of fitting procedure performed for dependences of imaginary part of complex dielectric function (*ε″*) versus frequency at various temperatures for poly(MEO_2_MA) samples of different architecture. Temperatures were selected arbitrarily to show all observed processes clearly. Additionally, for the highest temperature (343 K), the dependences of real part of complex dielectric function (*ε′*) versus frequency are presented to confirm presence of the α’ process.

Figure A1 presents examples of fitting procedure performed on DRS spectra obtained at various temperatures for linear poly(MEO_2_MA) and its mother and daughter networks. At the highest temperatures (bare dielectric spectra without fitting of particular processes are shown in Fig. 3 in the manuscript) the α process related to the segmental motions is well visible. Analysis of the real part of complex dielectric function (*ε′*) dependences on frequency reveals additional high frequency process (marked as α’) for all investigated samples. Although this process is masked by strong ionic conductivity in the dielectric loss dependences, there is a good agreement in the positions of the maximum of *ε”* and the inflection point in *ε′* curves, what proves the correctness of the applied fitting procedures.

The secondary processes observed in the glassy state were not analyzed in details in this work. As far as it was possible to separate them from each other using the fitting procedure, they exhibit similar behavior in all investigated systems, indicating that the sub-*T*_g_ processes are not, or one only weakly, affected by the network formation. The β–process shown in the center panel of Figure A1 is very weak, yet it turned out that using only three Havriliak-Negami functions (for processes α, γ and δ) was insufficient to fit correctly the dielectric spectra; for this reason one can postulate the presence of an additional, β–process. Processes δ and γ correspond to secondary relaxations and are best separated at the lowest temperatures (top panel). In this temperature range the processes α and β cannot be separated from each other and thus they are shown in top panel as the composite process α+β.


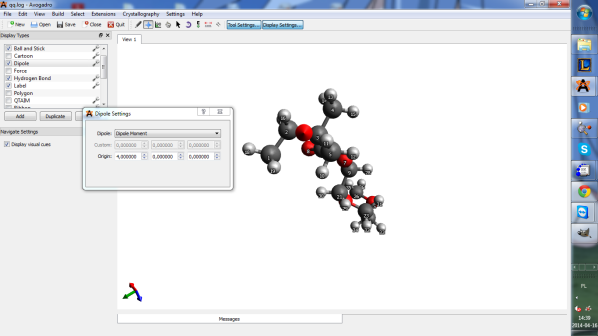

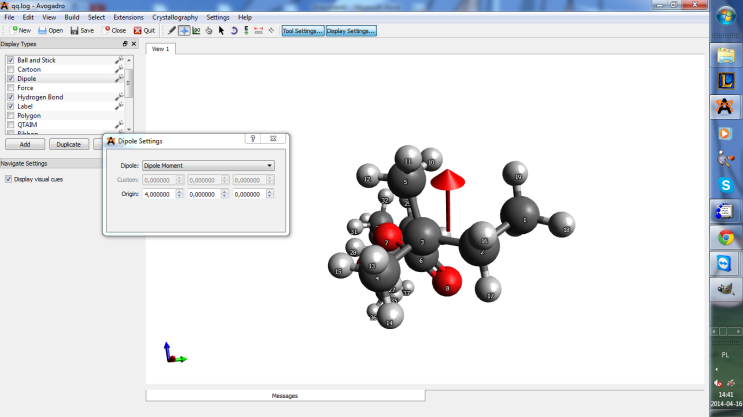


**Fig. A2** Two different projections on the optimized structure of 2-(2-methoxyethoxy)ethyl 2,2-dimethyl butanoate representing monomer unit of poly(MEO_2_MA). The resultant dipole moment is marked by red arrow. All trans configuration of main chain is marked by blue dots line, while the trans-gauche configuration by green dashed line.

Figure A2 presents structure of 2-(2-methoxyethoxy)ethyl 2,2-dimethyl butanoate optimized at the DFT/B3LYP level of theory expressed in the 6-31G(d,p) basis set, as implemented in the Gaussian 09 package. This molecule may represent the monomer unit of poly(MEO_2_MA). As it is commonly done, the long chains surrounding the monomer unit were replaced by methyl groups. The resultant dipole moment is marked in Figure A2 by a red arrow. Molecule selected as a model of poly(MEO_2_MA) monomer unit allows to consider two different configurations of the main chain: all trans (shown by blue dots line) and trans-gauche (shown by green dashed line). As one can see, in a case of the all trans configuration the dipole moment is perpendicular to the main chain. However, in the trans-gauche configuration, projection of the dipole moment onto the axis related to some aliphatic segments (marked by green continue line) constituting the main chain is nonzero. This result supports the hypothesis that the α’ process, seen both in the DRS and DMA spectra, can be regarded as the sub-Rouse mode.

**Fig. A3** Temperature dependences of dielectric strength (*Δε*) for linear poly(MEO_2_MA), mother and daughter networks. Open symbols corresponds to α, while the full ones to α’ processes respectively.

The temperature dependences of dielectric strength (*Δε*) for all investigated materials for α and α’ processes are shown in Figure A3. It is well visible, that the dielectric strength for α relaxation slightly decreases with increasing temperature for all investigated systems. It is consistent with literature data [A1] and characteristic for primary relaxations. It is also necessary to write that the values of *Δε* are similar for all samples. In a case of α’ relaxation the *Δε* also decreases with increasing temperature. The slope of the *Δε* = *f(1/T)* plot is the highest for linear polymer. For poly(MEO_2_MA) networks this dependence is very weak. The highest values of dielectric strength were found for daughter network, while the lowest one for linear polymer. It means that in a case of non-crosslinked poly(MEO_2_MA) macromolecules the correlation between the elementary dipole moments is the weakest one. Moreover, it strongly decreases with temperature. The highest value of *Δε* for daughter networks probably results from the presence of dangling chains. Taking into account the results of computer calculations presented in Figure A2, showing that the trans-gauche conformation of poly(MEO_2_MA) characteristic for helical topology of macromolecule results in non-zero dipole moment, it may be stated that significant part of dangling chains takes a helical conformation. Such scenario is highly probable, as the samples were obtained by drying and the flexible grafted chains were closed in confined space limited by network skeleton.

**Fig. A4** Dynamic temperature ramps (heating) for mother (blue open symbols and +) and daughter (green full symbols and x) networks. Circles corresponds to *G’*, stars to *G”*, while the crosses to *tan δ*. The results were recorded at the frequency 63 rad/s. Cooling/heating rate was 2 K/min.

The DMA results obtained for linear poly(MEO_2_MA) and presented in manuscript in Figure 4a unambiguously showed the presence of both α and α’ processes. Data collected for the networks are not so clear because of overlapping of discussed relaxations – see Figures 4b and 4c in the manuscript. Nevertheless the dynamic temperature ramps acquired for the mother as well as for the daughter samples and presented in Figure A4 prove beyond reasonable doubt that there are two relaxation processes in poly(MEO_2_MA) networks above the thermodynamic glass transition.

**Fig. A5** Activation map for poly(MEO_2_MA) materials differing on polymer architecture, constructed basing on DRS and DMA results (Figure 7 in the manuscript) with additionally shown (stars) characteristic relaxation times *τ_σ_* related to ionic conductivity determined as a point of intersection of *ε’* and *ε”* curves.

Figure A5 shows the temperature dependence of characteristic relaxation time *τ_σ_* related to ionic conductivity for all investigated poly(MEO_2_MA) materials in comparison with the high temperature relaxations (α and α’). Characteristic relaxation times corresponding to ionic conductivity at different temperatures were determined as point of intersection of *ε’* and *ε”* dependences vs. frequency. For linear polymer as well as for daughter network two regimes may be easily distinguished. Both may be fitted with VFT equations. At high temperatures the *τ_σ_(1/T)* closely correlates with the temperature dependence of the relaxation time of the α’ process, as both curves overlap. In the range of lower temperature (below ca 320-325 K) the discussed dependences bifurcate. For mother network similar tendency is observed, however it is impossible to separate mathematically specific regimes in the *τ_σ_* = *f(1/T)* curves.

[A1] Schlosser E., Schönhals A., Carius H.-E., Goering H. (1993) Evaluation Method of Temperature-Dependent Relaxation Behavior of Polymers. Macromolecules 26:6027-6032. doi: 10.1021/ma00074a027
